# The relationship between sleep and depression and bipolar disorder in children and young people

**DOI:** 10.1192/bjo.2021.1076

**Published:** 2022-01-14

**Authors:** Monica Comsa, Kirstie N. Anderson, Aditya Sharma, Vanishri C. Yadav, Stuart Watson

**Affiliations:** Child and Adolescent Mental Health Service, Cumbria Northumberland, Tyne and Wear NHS Foundation Trust, UK; Neurology Service, Newcastle upon Tyne NHS Foundation Trust, UK; Translational and Clinical Research Institute, Newcastle University, UK; and Child and Adolescent Mental Health Service, Cumbria, Northumberland, Tyne and Wear NHS Foundation Trust, UK; Child and Adolescent Mental Health Service, Cumbria, Northumberland, Tyne and Wear NHS Foundation Trust, UK; Translational and Clinical Research Institute, Newcastle University, UK; and Specialist Services, Cumbria, Northumberland, Tyne and Wear NHS Foundation Trust, UK

**Keywords:** Bipolar affective disorders, sleep disorders, depressive disorders, cognitive–behavioural therapies, comorbidity

## Abstract

**Background:**

Sleep difficulties are often reported in practice, and are part of the diagnostic criteria for depression and bipolar disorder.

**Aims:**

To inform the understanding of the relationship between sleep and both depression and bipolar disorder.

**Method:**

We conducted a narrative literature review of affective disorders and sleep difficulties in children and young people.

**Results:**

Specific sleep disorders, such as parasomnias, narcolepsy and sleep-related movement disorders, are associated with depression, whereas insomnia, obstructive sleep apnoea and circadian rhythm disorders are associated with both depression and bipolar disorder in children and young people. Conversely, children and young people with depression can present with a number of sleep difficulties, and these are associated with higher depression severity and greater fatigue, suicidal ideation, physical complaints, pain and decreased concentration. Sleep disturbances among adolescents with bipolar disorder can affect the severity of depressive and manic symptoms, are a poor prognostic indicator and have been associated with social and academic impairment. Antidepressants and antipsychotics can directly affect sleep architecture, which clinicians need to be aware of. Non-pharmacological interventions for sleep problems could prevent and/or minimise the risk of relapse in affective disorders.

**Conclusions:**

Sleep difficulties can occur before, during and after an episode of depression or bipolar disorder, and have a higher prevalence in affective disorders compared with the general population. A multi-modal approach would include the treatment of both the affective and specific sleep disorder. Further research is needed in this field to understand the impact of combined interventions on clinical outcomes.

## Introduction

Sleep is a biological necessity, essential for infant, child and adolescent growth; metabolism and immune system regulation; and normal memory and affect.^1,2^ This narrative review aims to serve as a clinician's guide, and will describe the following in children and young people: normal sleep, sleep disorders, the effect of affective disorders on sleep, the effect of disturbed sleep on mood and the role of targeted sleep therapy, particularly with respect to mood. Awareness of this complex relationship among healthcare professionals working with children and young people could lead to better care and improved long-term outcomes. This review does not discuss the relationship between sleep and affective disorders in children and young people with a neurodevelopmental disorder, such as hyperkinetic disorders, autism spectrum disorder and/or intellectual disabilities.

## Normal sleep

**Fig. 1:**
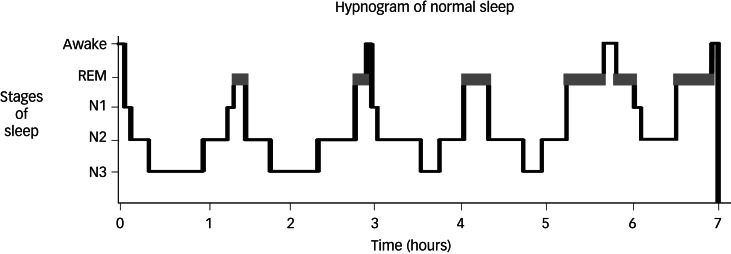
Hypnogram showing normal sleep in an adult. Non-rapid eye movement sleep is split into stages N1–N3. Courtesy of Dr Kirstie N. Anderson. REM, rapid eye movement.

**Fig. 2:**
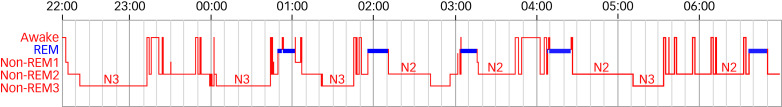
Hypnogram of a 9-year-old boy with a short sleep onset latency and shorter sleep cycle duration. Non-rapid eye movement sleep is split into stages N1–N3. Courtesy of Dr Elizabeth A. Hill, Royal Hospital for Sick Children, Edinburgh.

**Fig. 3:**
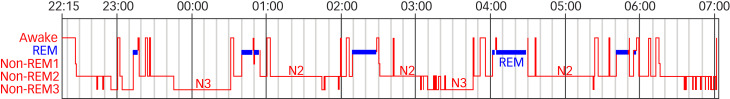
Hypnogram of a 14-year-old male. Longer (more adult-like) sleep cycle duration (some sleep fragmentation). Non-rapid eye movement sleep is split into stages N1–N3. Courtesy of Dr Elizabeth A. Hill, Royal Hospital for Sick Children, Edinburgh. REM, rapid eye movement.

Sleep is composed of two phases: rapid eye movement (REM) and non-rapid eye movement (NREM).^3,4^ NREM sleep is further divided into stages N1, N2 and N3, with stage N3 known as slow-wave sleep (SWS). REM sleep is associated with inhibition of peripheral muscle tone, along with active cortical function and increased blood pressure, heart and respiration rates.^1^ The switches that result in the cycling between REM and NREM are facilitated by monoaminergic and cholinergic neurons within the brain-stem (see Figs 1–3).^5^ Sleep is essential in the development of neurosensory, memory and motor systems in the foetus and neonate, and in the maintenance of brain plasticity over the lifetime.^2^ The overall regulation of sleep depends on two major processes: the sleep–wake homeostatic process and the circadian timing system. The homeostatic process ensures that the longer a person stays awake, the more pressure there is to fall asleep.^6^

## Sleep during childhood and adolescence

The duration of sleep, the duration of sleep cycles and the circadian rhythm differ between individuals, and vary during a person's lifetime. Newborns sleep most of the day and toddlers sleep for up to 12 h during the night, as well as having one or two naps during the day.^7^ REM and NREM cycles occur every 50–60 min in children under the age of 8 years, and these cycles gradually change to every 90 min as the child develops. Night waking is normal for all children, but is typically very brief, with rapid return to sleep. The proportion of stage N3 SWS is higher and the amplitude of the delta waves is far larger in children compared with adults; this proportion continuously reduces throughout adolescence and into adulthood (see Figs 2 and 3).^1^ During the final stages of puberty, there is a biological change in the circadian system whereby children fall asleep later and increase their total sleep time. This often coincides with a desire to stay up late at night to engage in more adult social activities. This delay in circadian phase leads to a delay in sleep onset,^8^ and may cause a lack of synchronicity between adolescent circadian rhythm and that of the parents and of societal structures (such as school timetables). These changes can lead to insufficient sleep, family discord and subsequent emotional difficulties.^9^ Sleep deprivation, particularly REM deprivation, in neonates and infants may have a permanent negative effect on development of neural circuitry of the primary sensory system, and of emotion, social learning and memory.^2^

## Affective disorders in adolescents

Major depressive disorder (MDD) in adolescence is characterised by a pervasive and persistent low or irritable mood^10^ and/or anhedonia, which can also be accompanied by low self-esteem.^11^ Adolescents with depression may report certain symptoms more often than adults, such as loss of energy, weight and appetite changes, and sleep difficulties, whereas adults report more anhedonia and concentration difficulties.^12^ Early and accurate detection and management of MDD is important because early onset can be a potent predictor of lifelong recurrent depression.^13^

Bipolar disorder is a chronic, typically relapsing condition, characterised by episodes of (hypo)mania and depression with a negative effect on overall functioning. In the DSM-IV^14^ and DSM-5,^10^ it has been separated into type 1, type 2, cyclothymia and not otherwise specified.^10,14,15^ A number of specifiers have been used for bipolar disorder when it occurs in young people, including prepubescent, juvenile, childhood or adolescent bipolar disorder.^10^ Bipolar disorder in adolescence is more likely to exhibit rapid cycling and mixed states, and the symptoms are more likely to have a fluctuating intensity and duration, thereby reducing the likelihood of accurate diagnosis compared with adults.^10^

Adolescent-onset MDD and bipolar disorder often have a chronic, episodic course, and can be accompanied by long-term functional impairment.^16^ Those affected have higher rates of substance misuse, poor academic attainment, interpersonal difficulties, poor sleep patterns and higher risk of suicide compared with healthy adolescents. These difficulties can negatively affect physical, emotional, cognitive and social development.^16–18^

## Sleep disorders

The International Classification of Sleep Disorders, Third Edition (ICSD-3)^19^ defines more than 70 sleep disorders classified into seven major categories, one of which is insomnia.^19^ The DSM-5^10^ describes ten sleep–wake disorders, and the ICD-10^11^ groups nonorganic sleep disorders into dyssomnias, parasomnias and nonpsychogenic disorders (see Table 1). When considering disordered sleep, it is worth noting that subjectively reported sleep correlates poorly with objective findings.^20,21^ For further information on specific sleep disorders, please refer to Table 2.

**Table 1:** Diagnostic entities in ICSD-3, DSM-5 and ICD-10

ICSD-3		DSM-5	ICD-10
Over 70 disorders classified in seven categories^19^		Ten disorders for adult, geriatric and paediatric patients^10^	Two categories of nonorganic sleep disorders in adults and children and one category of nonpsychogenic disorders^11^
		Category: sleep–wake disorders	Category: dyssomnias
Insomnia Sleep-related breathing disorders Central disorders of hypersomnolence Circadian rhythm sleep–wake disorders Parasomnias Sleep-related movement disorders Other sleep disorders		Insomnia disorder Hypersomnolence disorder Narcolepsy Breathing-related disorders Circadian rhythm sleep–wake disorders NREM sleep arousal disorders Nightmare disorder REM sleep behaviour disorder Restless leg syndrome Substance-/medication-induced sleep disorder	Insomnia Hypersomnia Nonorganic disorder of sleep–wake schedule
			Category: parasomnias
			Sleepwalking Sleep terrors Nightmares Other nonorganic sleep disorders Nonorganic sleep disorder, unspecified
			Category: nonpsychogenic disorders
			Disorders of initiating and maintaining sleep Disorders of excessive somnolence Disorders of the sleep–wake schedule Sleep apnoea Narcolepsy and cataplexy Other sleep disorders Sleep disorder, unspecified Episodic movement disorder

ICSD-3, International Classification of Sleep Disorders, Third Edition; NREM, non-rapid eye movement; REM, rapid eye movement.

**Table 2:** Sleep disorders

Name	Diagnostic criteria	Considerations	Treatment
Insomnia	DSM-5^10^ Dissatisfaction with sleep quantity or quality, with complaints of difficulty initiating and/or maintaining sleep, accompanied by clinically significant distress or impairment in social, occupational or other important areas of functioning, which can occur independently or during the course of another mental disorder or medical condition ICSD-3^19^ Persistent difficulty with sleep initiation, duration, consolidation or quality that occurs despite adequate opportunity and circumstances for sleep, and results in some form of daytime impairment. Subcategorised into chronic insomnia disorder, short-term insomnia disorder, other insomnia disorder, isolated symptoms and normal variants, excessive time in bed and short sleeper^19,22^	Paediatric insomnia Reports from young people and parents or carers need to be taken into consideration when assessing for insomnia^23^	Psychoeducation Behavioural management Pharmacotherapy
CRDs	Delayed sleep phase disorder Most common Typical sleep onset and wake time are more than 2 hours later than the age-related norm Causes difficulties waking up for school and lack of sleep Advanced sleep–wake phase disorder People tend to fall asleep and wake up more than 2 hours before their desired time Less common in those aged <18 years Irregular sleep–wake rhythm disorder Inconsistent sleep pattern	People with CRDs are unable to sleep and wake at the times required for normal work, school or social needs^11^	Sleep–wake scheduling Timed light exposure Melatonin
Sleep-related breathing disorders	OSA Snoring, unusual sleeping positions (e.g. hyperextended neck or seated with open mouth), sleep-related paradoxical breathing, night-time diaphoresis or enuresis, morning headaches, prolonged sleep time, difficulty waking, irritability and excessive daytime sleepiness	OSA is the most common sleep-related breathing disorder Affects 1–5% of children between 2 and 8 years of age^24^	Adenotonsillectomy is the primary treatment of this condition in those aged <8 years^25^ Continuous positive airway pressure therapy for older children^25^
Parasomnias	NREM parasomnias Confusion, automatic behaviours, difficulty awakening, minimal recall and often return to sleep after the event Sleepwalking More common in children than adults Can be associated with other parasomnias No distress to the child, but disrupts others^26^ Sleep terrors Episodes of partial, abrupt awakening from deep sleep, accompanied by inconsolable screaming and crying and autonomic arousal, but again, minimal recall for the child^10^ REM-related parasomnias Nightmares Typically occur in the last half of the sleep period^1,27,28^	NREM parasomnias Most common in children and are more likely in the first third of the night, during stage 3 sleep	Psychoeducation Behavioural management Treatment of comorbidities
		REM-related parasomnias Frequently encountered in childhood Often resolve in adolescence Common for all children but typically occur with recall and distress	
Narcolepsy and hypersomnia	Narcolepsy Excessive daytime sleepiness, fragmented night sleep, sleep paralysis, vivid dreams and hypnagogic hallucinations	Narcolepsy Rare sleep disorder resulting from the loss of a specific population of hypocretinergic neurons It has a prevalence of 0.1%^19^ and the mean age of onset is 14 years About 70% of affected people have cataplexy	Psychoeducation Behavioural management Pharmacotherapy (stimulants)
	Primary hypersomnia in children Excessive sleep duration for developmental norms, impaired quality of wakefulness or sleep inertia^29^	Primary hypersomnia in children Has a prevalence of 0.8%^29^ May simply represent a slight delay in normal development^30^	

ICSD-3, International Classification of Sleep Disorders, Third Edition; CRD, circadian rhythm disorder; OSA, obstructive sleep apnoea; NREM, non-rapid eye movement; REM, rapid eye movement.

### Insomnia

The diagnostic criteria for insomnia in adults and young people are the same; however, in the younger population, the problems around initiating and maintaining sleep can also be reported by the carers. These difficulties are typically associated with bedtime resistance and behavioural change.^23^

### Circadian rhythm disorders

The social zeitgeber theory posits that changes to the circadian rhythm can be linked to disruptions of bedtime, wake time or meal times.^31^ Circadian rhythm sleep disorders (CRDs) are caused by desynchronisation between the internal sleep–wake rhythms and the light–dark cycle, or a misalignment between the body clock and the person's external environment.

### Sleep-related breathing disorders

Among the sleep-related breathing disorders, obstructive sleep apnoea (OSA) is the most common and is primarily caused by enlarged tonsils and adenoids.

### Parasomnias and nightmare disorders

Parasomnias are divided into two categories: REM and NREM. They affect up to 50% of children^27^ and are a risk factor for the subsequent development of MDD in adulthood.^32^ REM-related parasomnias, such as sleep paralysis or nightmare disorder, are frequently encountered in childhood and, like NREM parasomnias, they often resolve spontaneously in adolescence.^1,27,28^

### Narcolepsy and hypersomnia

Narcolepsy is a rare sleep disorder caused by the loss of a specific population of hypocretinergic neurons. This condition has been associated with depression both in adults^32^ and the paediatric population.^33–35^ Primary hypersomnia may be transient or may represent a slight delay in normal development,^30^ and has not been associated with affective psychiatric disorders in adulthood. Bouts of episodic hypersomnia, along with cognitive and behavioural changes, are part of the very rare condition of Kleine–Levin syndrome.

### Sleep-related movement disorders

Sleep-related movement disorders, such as restless legs syndrome (RLS), periodic limb movement disorder (PLMD) or the far less common condition of rhythmic movement disorder, are conditions that cause movement during or before sleep.

### Sleep disorders and their relationship with mood

Behavioural pattern insomnia occurs more often in children and has been associated with symptoms of depression in preschoolers.^29^ It is important to be mindful of the parent–child interaction around bedtime, and a thorough assessment often helps to identify the aetiology of the sleep problem. Children can become habituated into needing a bedtime story or other event as the associated condition to fall asleep. This can create sleep difficulties, such as when children wake through the night and are unable or unwilling to self-settle in its absence. Sleep difficulties can occur when parents struggle to implement boundaries or routines around bedtime in older children. Additionally, certain parental expectations that are not in line with the developmental stage^36^ and children's sleep needs might lead to their erroneous identification of disordered sleep, in particular the natural later bedtimes through puberty and teenage years.

Data from school-aged children^37^ and adult research have shown an association between OSA and depression.^32,38^ OSA is more prevalent in people with bipolar disorder, both in adults^39^ and adolescents,^40^ but research in this field remains sparse.

Significant, intrusive and distressing parasomnia has been associated with suicidal thinking^41^ and, in younger children (aged 12 years), with psychotic phenomena, albeit rarely.^42^ Adults with MDD and bipolar disorder are more likely to have experienced parasomnias, such as confusional arousal disorder, night terrors and sleepwalking, than the general population, but the nature of this association remains unclear given how common these symptoms are in children and are mostly benign, infrequent and self-limiting.^43^

When frequent and intrusive, nightmares can occasionally result in daytime impairment and reduced quality of life, and are associated with psychological distress and symptoms of anxiety and depression.^44,45^ Sheaves et al found that high nightmare frequency followed by subsequent distress was positively associated with higher scores in difficulties, such as depression, among students.^46^

Narcolepsy has been associated with depression in adults^32^ and in the paediatric population.^33–35^ Additionally, there are no biomarkers for Kleine–Levin syndrome, its aetiology is debated, there is spontaneous remission and one study suggested that young people with a history of Kleine–Levin syndrome may experience depression later in life.^47^

CRDs have a higher prevalence in adults with bipolar disorder than the general population,^48,49^ with a significant association with a younger onset of bipolar disorder.^50^ CRDs are also risk factors for developing MDD in adults,^32^ and have been associated with depressive symptoms in adolescents.^51^

RLS and PLMD have been associated with depression in adults when severe and sleep disruptive.^32^ In children and young people, RLS has a prevalence of 2–4%^52^ and has been associated with depression, although to a lesser extent, and usually the condition is milder in younger adults.^53^ Adolescents with PLMD also appear to display more depressive symptoms.^54^

## Mood disorders and their relationship with sleep

Disturbed sleep is a core feature of a depressive episode,^55^ may occur in the prodromal period^56,57^ and/or be a residual symptom of depression.^55^ Conversely, mood disturbance is listed as one of the functional impairment criteria for insomnia disorder.

In the general teenage population, insomnia is associated with mental health difficulties later in life.^58^ Disturbed sleep confers an increased risk of subsequent bipolar.^59–63^ Wakefulness in bed^56^ and a shorter duration of sleep^57^ can be regarded as aetiological factors for depression.^64–67^ Sleep difficulties can precede affective episodes^59,61–63,68,69^ and other mental health difficulties^58^ by several years in young people. Aspects of the sleep architecture of insomnia, such as reductions in SWS, low spindle activity or changes in REM, are also associated with internalising behaviours.^70^

In young people who are currently depressed, disturbed sleep incorporating sleep continuity problems,^17^ wakefulness in bed and increased sleep-onset latency is common. Decreased sleep efficiency, short sleep duration, daytime sleepiness, non-restorative sleep, hypersomnia and irregular sleep–wake rhythm are also common.^12,17,20,29,56,57,71–75^ Sleep difficulties are associated with higher depression severity and recurrence;^76^ greater fatigue, suicidal ideation, physical complaints and pain;^17^ and decreased concentration.^73,77^ There is evidence that sleep quality, total sleep time and sleep microarchitecture can predict new onset and recurrence of depression.^78^

Robust adult data and preliminary data in young people suggests that sleep problems can be encountered in all phases of bipolar disorder,^79^ can be associated with psychosocial impairment and can play a role in predicting mania and depression.^80–82^ Sleep disturbances among adolescents with bipolar disorder can have an effect on the severity of depressive^80,81^ and manic symptoms, are associated with academic and social impairment during recovery,^80^ and can be a poor prognostic indicator. Young people with bipolar disorder show a range of sleep difficulties, including delayed sleep onset, insomnia, hypersomnia and difficulties waking up in the morning. They can also present with difficulties maintaining a regular rhythm, longer naps during the day, more night-time wakings, a greater total time awake during the night and nightmare disorder.^80,83–86^ Nightmares appear to augment the risk of suicide, and insufficient sleep duration has been associated with self-injurious behaviours.^84^ Mania has been associated with decreased need for sleep in young people;^87^ however, in adolescents, mania also appears to be linked to a broader sleep disturbance,^59^ including variable sleep duration and unstable morning routines.^80^ Sleep disturbance does not appear to differentiate between types of bipolar disorders, being as common, for instance, in bipolar disorder not otherwise specified as in bipolar disorder type 1.^88^ Disturbed sleep is seen more often in the children of parents with bipolar disorder than in healthy controls.^61,89^ One study of children whose parents were diagnosed with bipolar disorder showed shorter time to onset of sleep and longer duration on actigraphy, with opposite subjectively reported experiences.^90^ This is in accordance with a second study showing that people deemed to be at high risk of developing bipolar disorder, either through family history of bipolar disorder or personal history of severe depression and subthreshold mania symptoms, have shown a longer duration for sleep onset, but also higher levels of sleep specific worries.^85^ Depressed youth with bipolar disorder are significantly more likely to be affected by daytime sleepiness and hypersomnia than young people with unipolar depression.^86^

## Mechanism of the association between mood disorders and sleep

Maladaptive thinking processes, such as cognitive inflexibility, attention bias by selectively focusing on negative information, misperception of sleep deficit, rumination and worry, may underlie insomnia, depression and bipolar disorder in adolescence.^70^ These processes may trigger autonomic arousal and the reinforcement of negative cognitions. Similarly, abnormalities of circadian rhythm, perhaps mediated by genetic vulnerability via polymorphisms of serotonin, dopamine and circadian clock genes^60,91^ or dysfunction in the white matter integrity, could also play a role in the aetiology of these conditions.^92^ Disruption of the corticolimbic circuitry^70^ may be a consequence of insomnia, and may impair affective reactivity and regulation.^93^ Dysregulation of reward/approach related brain function,^70,94^ hypothalamic-pituitary-adrenal axis dysregulation and elevated inflammatory cytokines may also contribute to both psychopathology and sleep disturbance.^70^ Disruptions to the circadian rhythm have been postulated to contribute to the pathophysiology of bipolar disorder.^95^ Genetic commonality between sleep and impulsivity and anger/frustration has also been described.^96^ Antidepressants, particularly those with noradrenergic or dopaminergic mechanisms, may induce or aggravate insomnia. Electronic media use among adolescents, particularly at night-time, may be related to sleep disturbance and higher levels of depressive symptoms,^97^ in which a sleep debt developed over school days is paid back at the weekend.^98^

## Interventions for mood disorders and sleep problems

### Non-pharmacological interventions for depression and sleep problems

Psychoeducation, consistent sleep–wake schedules and tailored interventions for addressing sleep problems should play an important role in the prevention and treatment of mental health difficulties, at any age. Concomitantly addressing sleep problems can lead to an increased remission rate of depression.^67,99^

Cognitive–behavioural therapy for insomnia (CBT-I) has a strong evidence base for decreasing insomnia severity.^70,100^ There have been a number of studies of CBT-I in young people with comorbidities (e.g. Moore et al^101^); two randomised controlled trials in young people not selected on the basis of comorbidity have shown a persistent benefit in sleep onset, latency, efficiency and anxiety, but not in total sleep time.^102,103^ Internet-delivered CBT-I has also been examined. Recent large, randomised controlled trials, one of which targeted university students,^104^ have shown a benefit in sleep and a number of mental health problems, such as depression, anxiety and psychotic phenomena.^104,105^

Transdiagnostic sleep and circadian intervention collates established sleep management strategies,^106^ and has been shown to exert a sustained beneficial effect on eveningness circadian preference in adolescents.^107^ *Post hoc* analysis revealed that the initial broader health benefits were not maintained (compared with psychoeducation) at 6 months.^107,108^

### Pharmacological intervention for depression and sleep problems

Many patients with depression enter REM stage quicker and have less NREM sleep in the first sleep cycle. Antidepressants typically alter sleep in the opposite direction.^109^ Selective serotonin reuptake inhibitors (SSRIs) are the antidepressants of choice in children and young people. It is important that sleep effects are considered in the discussion of positives, negatives and gaps in knowledge that are required to allow an informed decision of whether and when to use medication alongside psychosocial interventions for treating depression. SSRIs are known to increase REM latency and SWS, reduce REM sleep and sleep continuity, and may also induce nightmares, cause enhanced dreaming and lead to changes in dream content.^65,109–114^ Upon stopping the treatment, rebound REM sleep is known to occur.^110^ SSRIs can increase the risk of aggravating RLS, or induce RLS or PLMD symptoms.^115^

### Melatonin

Melatonin is often used for children with sleep-onset insomnia or delayed sleep phase syndrome and neurodevelopmental disorders. High doses of melatonin are rarely needed for managing insomnia. Behavioural strategies should be used as a first-line treatment.^116–118^ Adults with affective disorders have been found to have abnormalities in the timing and amplitude of biological rhythms, including abnormal patterns of melatonin secretion.^119^ Differences in the level of melatonin and in the pattern of its production have been described in adults with bipolar disorder across mood states.^120^ A recent study suggested that melatonin levels are related to social and occupational functioning in young people with affective disorders. The putative role of melatonin in the treatment of depression in young people is an exciting prospect.^121^

### Non-pharmacological interventions for bipolar disorder and sleep

Regulating sleep and maintaining consistent sleep–wake schedules in young people with bipolar disorder improves outcomes, and potential affective relapses can be prevented and/or minimised. This may be enacted via psychoeducation, the development of consistent sleep and wake times and routines, relaxation procedures, and reinforcement of good sleep habits,^122^ chronotherapy^123^ or CBT-I.^124^

### Pharmacological interventions for bipolar disorder and sleep

The use of psychotropic medication in children and young people is only advisable when a clear diagnosis of bipolar disorder has been made, and should not be used solely for addressing sleep. Improved sleep is associated with pharmacologically mediated improvement in mood symptoms in adolescents,^84^ but it is worth considering whether the effect of bipolar psychotropics on sleep mediates some of these changes. Olanzapine and quetiapine have a significant impact on sleep duration in younger people with bipolar disorder.^85^ Antipsychotics, including olanzapine, quetiapine and ziprasidone, have been shown in the adult population to increase the duration of sleep continuity, increase total sleep time and sleep efficiency, increase REM latency and SWS, decrease stage 2 sleep spindle density and reduce the amount of REM sleep.^125^

The increased risk of metabolic syndrome in bipolar disorder also increases the risk of OSA in adolescents.^40^ Another study suggested a possible relationship between the use of medication and enuresis,^88^ which could further affect the sleep.

## Research direction

There are a number of challenges in the field of sleep research in MDD and bipolar disorder. First, most studies are cross-sectional, with an unclear timeline between the onset of the affective disorder, that of sleep difficulties and the short- and long-term complications.^83^ There is still limited objective sleep assessment with polysomnography, and many studies report subjective sleep via self-/parent report or actigraphy without, for example, screening for specific sleep disorders or using polysomnography. The parent–child agreement for psychiatric disorders is low and, wherever possible, the children and young people should be consulted about their sleep directly; depression and bipolar disorder are no exception.^84,126^ Terms such as sleep disorders and sleep difficulties are used interchangeably, and there is a need for greater precision with the use of, for example, insomnia disorder or CRD. Additionally, there seems to be a very high reliance on assessment questionnaires for diagnosing sleep problems and sometimes even affective disorders. Over the years, there have been inconsistent findings in the studies for depression in children and young people,^127^ as study designs vary a great deal.

The number of patients included in trials for bipolar disorder tends to be relatively low, which might be linked to the relatively low prevalence in children and adolescents and the subsequent recruitment challenge. Some studies seem to focus on one type of bipolar disorder and do not include the full spectrum of disorders, which might leave out important information.^88^ Findings vary across studies because of differences not only in the sleep measurement, but also sampling; some studies focus on children who have been diagnosed with bipolar disorder, whereas others include children whose parents have an affective disorder.^81^ There is also a high variability of the age range, as there is no consensus on the age of onset of paediatric bipolar disorder; some papers have included participants of 5 years of age,^88^ whereas others have focused on prepubescents^128^ or adolescents.^80^ One has to consider the importance of brain maturation and expected sleep changes that occur in these stages of life, as well as the beliefs around sleep within the family.

An important direction for future research would be developing a robust study design that allows a longer-term evaluation of the symptoms of a bigger cohort, objective and subjective standardised measures of sleep and mood, screening for comorbid sleep disorders, collecting collateral information and focusing on behavioural interventions for sleep, such as CBT-I.

## Conclusions

The relationship between sleep difficulties and mood in children and young people is complex. Sleep problems are reported long before the onset, during and sometimes after, an episode of unipolar depression or bipolar disorder, and can play an important role in their presentation. The prevalence of sleep disorders, such as insomnia, CRDs and sleep apnoea, in depression and bipolar disorder is higher than in the general population, but often these diagnoses are missed and specific treatment not offered. A multi-modal approach would include the treatment of both the affective and specific sleep disorder. ‘Sleep disturbance’ is not a diagnosis. There is a need for further longitudinal studies with subjective and objective sleep measures to improve therapy and the nature of the relationship between mood and sleep. Of most importance is the impact of specific treatment for sleep disorders on the long-term outcome of affective disorders. Clinicians need to know if better nights reliably lead to better days.

## Data Availability

Data availability is not applicable to this article as no new data were created or analysed in this study.

## Supplementary material

For supplementary material accompanying this paper visit https://doi.org/10.1192/bjo.2021.1076. click here to view supplementary material
